# Deciphering DNA Methylation in HIV Infection

**DOI:** 10.3389/fimmu.2021.795121

**Published:** 2021-12-02

**Authors:** Thilona Arumugam, Upasana Ramphal, Theolan Adimulam, Romona Chinniah, Veron Ramsuran

**Affiliations:** ^1^ School of Laboratory Medicine and Medical Sciences, College of Health Sciences, University of KwaZulu-Natal, Durban, South Africa; ^2^ Centre for the AIDS Programme of Research in South Africa (CAPRISA), University of KwaZulu-Natal, Durban, South Africa; ^3^ KwaZulu-Natal Research Innovation and Sequencing Platform (KRISP), School of Laboratory Medicine & Medical Sciences, University of KwaZulu-Natal, Durban, South Africa

**Keywords:** HIV, epigenetic regulation, DNA methylation, epigenome-wide methylation, epi-therapeutics

## Abstract

With approximately 38 million people living with HIV/AIDS globally, and a further 1.5 million new global infections per year, it is imperative that we advance our understanding of all factors contributing to HIV infection. While most studies have focused on the influence of host genetic factors on HIV pathogenesis, epigenetic factors are gaining attention. Epigenetics involves alterations in gene expression without altering the DNA sequence. DNA methylation is a critical epigenetic mechanism that influences both viral and host factors. This review has five focal points, which examines (i) fluctuations in the expression of methylation modifying factors upon HIV infection (ii) the effect of DNA methylation on HIV viral genes and (iii) host genome (iv) inferences from other infectious and non-communicable diseases, we provide a list of HIV-associated host genes that are regulated by methylation in other disease models (v) the potential of DNA methylation as an epi-therapeutic strategy and biomarker. DNA methylation has also been shown to serve as a robust therapeutic strategy and precision medicine biomarker against diseases such as cancer and autoimmune conditions. Despite new drugs being discovered for HIV, drug resistance is a problem in high disease burden settings such as Sub-Saharan Africa. Furthermore, genetic therapies that are under investigation are irreversible and may have off target effects. Alternative therapies that are nongenetic are essential. In this review, we discuss the potential role of DNA methylation as a novel therapeutic intervention against HIV.

## Introduction

In the nuclei of eukaryotes, the chromatin is subject to intense epigenetic events resulting in either condensed repressive heterochromatin or transcriptionally permissive euchromatin (1). These epigenetic events include posttranslational modifications to histones and methylation of DNA (1). DNA methylation involves the covalent addition of methyl groups to the fifth carbon in the nitrogenous base of cytosine (5mC) bases that are usually followed by guanine bases (CpG site) in DNA (2–5). Methylation of CpG sites found in the cis-regulatory regions of genes is generally associated with silencing genes (5–7). Methylation can also occur in intergenic regions, where it prevents the expression of potentially harmful genetic elements (4) as well as within the gene body, where a positive correlation with gene expression occurs (8–10).

DNA methylation is strongly involved in the physiological control of gene expression (4). It plays a key role in normal development (11), compaction of chromatin (12), genomic imprinting (13), X chromosome inactivation (14) and the bulk silencing of viral and transposable elements (15). However, aberrant methylation patterns are associated with a multitude of diseases [reviewed in (16–19)]. Several studies have shown that viral infections can induce aberrant methylation patterns within the host genome (20–22). On the other hand, the integrated proviral genome is also influenced by the epigenetic environment of the host (20, 23, 24). Thus virus-host interaction induces an altered epigenetic environment that affects both the virus and the infected host cell.

The human immune deficiency virus (HIV) is no exception to this phenomenon. The effect of HIV infection on DNA methylation has been characterised in HIV positive individuals (25). These effects have been associated with accelerated aging and abnormalities in gene expression, especially in immune regulating genes (25–30). Furthermore, methylation of HIV provirus by the host’s methylation machinery can control HIV-1 transcription, replication, and persistence (31–35).

We review the current literature on viral and human genes affected by methylation as well as address gaps in knowledge that are yet to be explored with regards to DNA methylation and HIV. This review will focus on five aspects: (i) the fluctuations of host DNA methylation modifying factors post HIV infection, (ii) the contribution of methylation on viral genes, (iii) the contribution of human genomic methylation on HIV disease, (iv) the influence of methylation on host genes observed in other diseases and models, and (v) the potential of DNA methylation as an epi-therapeutic strategy and precision medicine biomarker.

## DNA Methylation Modifying Factors Post HIV Infection

DNA methylation is not a random event. Several proteins are involved in establishing, removing, and recognising methylation marks at specific CpG sites within the eukaryotic genome (4). DNA methylation is established by a family of DNA methyltransferases (DNMTs – DNMT1, DNMT3a and DNMT3b). DNMT1 is responsible for maintaining methylation patterns following DNA replication (36), while DNMT3a and DNMT3b regulate *de novo* methylation (37). Therefore, alternations in DNMT expression usually leads to changes in DNA methylation levels within cells. Previous studies have highlighted the increase in expression of DNMTs in HIV infected CD4^+^ T cells (38–41). HIV-1 was shown to induce the expression of DNMT1 in a non-specific tissue manner, and that overexpression of the viral genes: *nef*, *tat* and *rev*, induced *DNMT1* promoter activity (40, 42, 43). In regulatory T cells, the effect of X4-tropic HIV infection demonstrated no significant change in the expression of DNMT1 and DNMT3a, while there was a substantial increase in expression of DNMT3b (41); however, increased expression of DNMT1, DNMT3a and DNMT3b was observed in CEM*174 T cells with significantly higher expression of DNMT3b (44). Similarly, HIV-1 replication enhanced DNMT3b levels in patients receiving antiretroviral therapy (ART) (45). The expression of DNMT3b was directly correlated to patient HIV viral load, while an inverse relation was observed for DNMT1 (45). Furthermore, proteomic analysis of primary oral epithelial cells revealed significantly lower DNMT1 and DNMT3a levels in HIV patients on ART. Additionally, DNMT activity and global DNA methylation illustrated a direct correlation (46).

The effect of HIV on DNMTs has incited interest in its effect on DNA demethylase enzymes. Conversion of the methyl group from 5-methyl-cytosine are mediated by a group of ten-eleven translocation methylcytosine dioxygenase (TET) enzymes to generate 5-hydroxymethyl-cytosine. 5-hydroxymethyl-cytosine can undergo further modifications such as deamination by apolipoprotein B mRNA Editing Catalytic Polypeptide-like (APOBEC) proteins. The expression of DNMT1 and TET1 was found to be increased in HIV-1 infected individuals without ART (47). Recently, the HIV-1 Vpr, which increases HIV-1 replication in macrophages, was shown to target TET2 for degradation, exacerbating HIV-1 infection (48, 49). The status of other TET enzymes (such as TET2 and TET3) has not been explored in an HIV setting.

Interestingly, recent studies have highlighted the importance of TET2 and TET3 for regulatory T cell stability and immune homeostasis (50). The loss of TET3 gene expression may be a pivotal contributor to locus hypermethylation (51). The effect of the TET family in an HIV setting is vastly unexplored; thus, the future investigation may unearth potential mechanisms of action, as seen in non-communicable diseases (52–54). However, much interest has been given to the cytidine deamination functioning of APOBEC (especially APOBEC3G and APOBEC3F). They have been shown to extensively deaminate viral cytosine to uracil resulting in the potent inhibition of HIV-1 infections (55, 56).

Another key multifunctional epigenetic regulator associated with HIV is methyl CpG-binding protein-2 (MeCP2), which recognizes methylated CpG sites and modulates transcription and chromatin structure (57, 58). The HIV gene *tat* is known to induce miR-132 expression, which subsequently down-regulates the expression of MeCP2 (59). However, Periyasamy et al. (60) discovered that the HIV-1 tat protein downregulated miR-124, which increased MeCP2 and its phosphorylated (Ser80) analogue in microglial cells. Interestingly, phosphorylated MeCP2 (Ser80) blocks miRNA biogenesis machinery, subsequently down regulating miR-124. These contradictory observations suggest that the effect of HIV-1 on host genes desires more attention (60).

DNA methylation is also known to be recognized by methyl-CpG binding domains (MBDs) and Ubiquitin Like with PHD and Ring Finger Domains 1 (UHRF1), which recruits DNA methylation modifying enzymes to chromatin (61, 62). Evidence from Kauder et al. (31) showed that HIV latency is regulated epigenetically *via* methylation of proviral DNA by DNMTs and its recognition by MBD2 (31). UHRF1 was also shown to facilitate latency as it was recruited to the HIV-1 5’LTR in a methylation/integration dependent fashion, where UHRF1 mediates the repression of HIV-1 gene expression (63).

## Contribution of Methylation on Viral Genes

**Figure 1:**
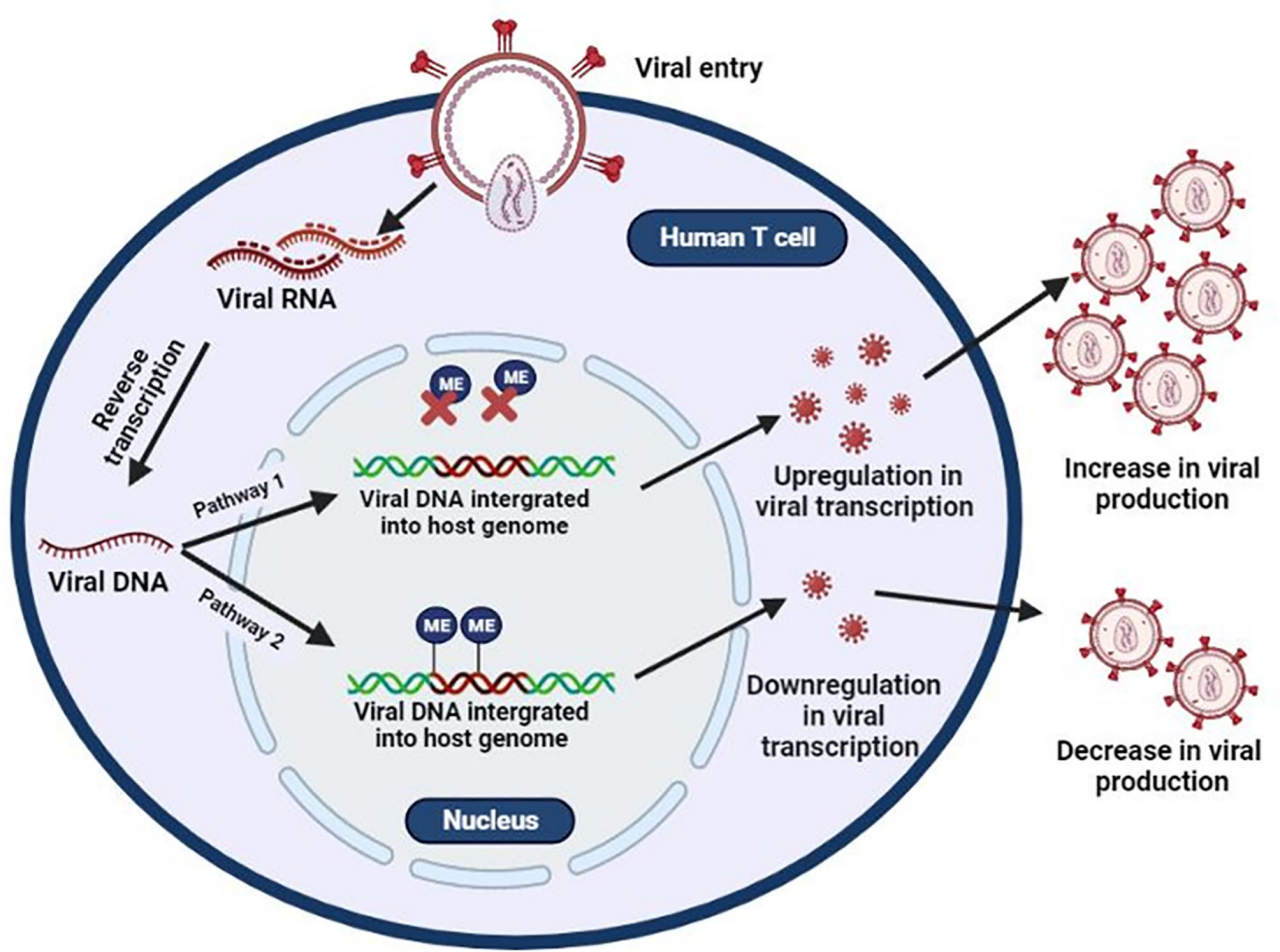
Epigenetic silencing of HIV transcription *via* methylation of integrated provirus. HIV binds to host receptors, entering the cell. HIV viral RNA is converted to single-stranded viral DNA and integrates into the host genome. HIV uses the host’s machinery to create new HIV copies (Pathway 1). However, methylation of integrated provirus results in the downregulation of HIV viral transcription, resulting in latency (Pathway 2).

Methylation of both the HIV-1 proviral genome and host genome facilitates the integration, replication, and latency of HIV-1. The integration of proviral DNA into the host chromosome is not random as it is preferentially inserted into the euchromatin or actively transcribing regions of the host (64–66). Once integrated, it becomes indistinguishable from the host genome and exploits host cellular machinery for the transcription of its genes (67). However, this also puts proviral DNA at risk for epigenetic silencing events such as DNA methylation. In most cases, presence of methylation within the viral DNA, which has been integrated into the host genome, results in the reduction of new viral particles. In contrast, when integrated viral DNA is not methylated, viral transcription and viral production proceeds as usual (**Figure 1**).

The association of proviral methylation and the transcriptional inactivation of HIV-1 was introduced as early as 1990 (68, 69). Since then, several *in vitro* studies have reported that methylation of CpG sites found within the proximal proviral promoter, located in 5’ long terminal repeat (5’LTR), silences transcription of HIV-1 genes resulting in latency. This allows HIV to evade host immune responses and ART (31–35). However, *in vivo* analysis of methylation patterns in the 5’LTR with regards to latency is conflicting. High methylation patterns were found in the 5’ LTR of memory CD4+ T cells isolated from aviraemic HIV positive individuals on long term ART therapy (32). However, in a subsequent study, CpG sites were poorly methylated in resting CD4+ cells from HIV infected individuals (70). Trejbalova et al. (33) also observed low methylation levels in the 5’LTR of resting CD4+ cells isolated from individuals on effective ART; however, methylation levels appeared to increase with prolonged ART use (33). A comparison of methylation levels in the 5’LTR of long term non-progressors/elite controllers and virally suppressed individuals on ART found that methylation was virtually absent in individuals in the latter groups compared to the non-progressor/elite controller groups (71). These observations suggest that latency and ART-induced suppression might have different methylation patterns. The apparent difference in methylation patterns between *in vivo* and *in vitro* studies can be attributed to the pressure of “natural” selection in HIV-1-infected individuals. In contrast, under *in vitro* cell culture conditions, HIV-1 proviral genomes are not subject to the selective pressure exerted by host immune defence (72).

5’LTR methylation levels were also shown to associate with the expression of HIV-1 genes. Decreasing levels of methylation in the 5’LTR corresponded with increasing expression of HIV-1 *gag* in HIV-1 infected spermatozoa. Furthermore, gag protein was expressed in 2 cell embryos transfected with infected spermatozoa suggesting that 5’LTR methylation regulates the expression of HIV-1 *gag* in the vertical transmission from sperm to embryo (73).

Regarding methylation patterns outside the LTR, Weber et al. (72) found that CpG sites remain in a predominantly unmethylated state in the 5’LTR, 3’LTR and portions of HIV-1 *gag, env, nef* and *tat* genes. They also observed slight variations in the methylation state of the HIV-1 genome in one long term non-progressor over 11 years, although viral load and CD4^+^ levels remained stable (72). A recent study examined methylation of intragenic regions of the proviral genome across four groups of HIV infected individuals [i.e. long term non-progressors, early combination ART (cART) treated, late cART treated and cART naïve, acutely infected] (74). As a whole, methylation of promoter regions was reduced in all four groups, while high levels of methylation were observed in the intragenic *env* region. In the ART naïve acutely infected group, a distinct increase in 5’LTR and a decrease in intragenic *env* methylation was observed (74). Taken together, these observations suggest that intragenic methylation could be a late event during infection as well as intragenic methylation was positively associated with CD4+ counts and viral loads (74).

It is important to take into consideration the high mutation rates of HIV (75). Based on levels of mononucleotide C and Gs, the frequency of CpG sites within the HIV-1 genome is much lower than one would expect. The methylation of viral CpG sites may result in the spontaneous deamination of cytosine to thymine which increases the mutation rate of HIV (76). Moreover, coding regions such as the *env* region are highly variable (75). It would be interesting if future studies would evaluate whether CpG sites within these regions are lost, retained, or gained over a period of time and whether these mutations are beneficial or harmful to the virus.

Thus far, all studies on HIV DNA methylation have focused only on CpG methylation; however, non-CpG methylation was reported in other retroviral infections (77, 78). The lack of appropriate techniques that include non-CpG methylation may be why it has not been evaluated, as most of the studies discussed used nested PCR-based methods that exclude most non-CpG methylation (79).

The variability in existing data may be due to several factors. For instance, the integrated HIV provirus is subject to its immediate chromatin environment; thus, different integration sites may influence methylation status accordingly (79). Several pitfalls arise from the amplification of HIV from bisulphite-converted DNA: (i) the high mutation rates of actively replicating HIV hinders designing PCR primers that can amplify all HIV targets, (ii) longer primers are needed for bisulphite converted DNA which can worsen the biased amplification of variable sequences, (iii) multiple rounds of amplification of multiple variants can introduce stochastic bias and variable results are obtained from different methods even when the same conditions are applied (79). There is a significant need for an approach in which HIV amplification of the provirus is reproducible across different primer sets and experiments. Furthermore, attempts to establish and measure latency are unconvincing. It has previously been shown that cell lines harbouring viruses are not genuinely latent but are instead in an incapacitated state (80). Thus, *in vitro* studies are not an accurate measurement of methylation or latency. The development of appropriate methods for specific assessment of the replication-competent HIV reservoir in clinical samples and techniques of studying DNA methylation in the context of HIV may be helpful. Furthermore, the examination of non-CpG methylation of the provirus should be undertaken (79).

## The Contribution Human Genomic Methylation on HIV Pathogenesis

While DNA methylation may influence the replication of HIV and transcription of crucial HIV genes, the integration of HIV-1 DNA into the host genome is also associated with aberrant methylation of host genes. Altered DNA methylation across the host genome has been shown to contribute to HIV disease. Previous studies have identified this *via* two different mechanisms. The first mechanism is a non-hypothesis driven approach which characterizes epigenome-wide methylation patterns. The second method is a hypothesis driven approach which measures methylation of specific/candidate genes. We will discuss each approach more thoroughly in the two sections which follows.

### Assessing the Epigenome-Wide Methylation Patterns of the Host

Given that methylation of specific CpG sites found in either the promoter or gene body may impact gene expression, the use of epigenome-wide characterisation of DNA methylation provides a powerful approach in identifying epigenetic variations associated with disease acquisition, severity, and predictive outcomes (81, 82). Several high-throughput methods have been established for the genome-wide profiling of methylation at single-nucleotide resolution. These methods usually require the treatment of genomic DNA with sodium bisulphite, which deaminates unmethylated cysteine residues to uracil, leaving methylated cysteine residues unaffected (83). The most used techniques include whole genome-wide bisulphite sequencing and microarrays. Whole genome-wide bisulphite sequencing involves PCR amplification of bisulphite converted DNA coupled with next-generation sequencing, which allows for the methylation profiling of every cytosine in the genome (84). Methylation arrays such as Illumina’s Infinium arrays involves amplifying bisulphite converted DNA followed by its hybridisation to arrays containing probes that distinguish methylated and unmethylated cytosine and covers CpG islands, shores, and shelves (85). Other methods include methylated DNA immunoprecipitation, comprehensive high-throughput arrays for relative methylation and reduced-representation bisulphite sequencing. Most of the studies pertaining to genome-wide methylation profiling in HIV infected hosts use either methylation arrays or whole genome-wide bisulphite sequencing.

The first large scale study to characterise altered DNA methylation patterns of the host genome associated with HIV infection was conducted on DNA extracted from whole blood collected from 261 HIV infected and 117 uninfected individuals (30). The epigenome-wide association study (EWAS) identified 20 CpG sites to be significantly associated with HIV infection. Among them, 14 CpG sites were found to be hypomethylated, and six were found hypermethylated in HIV-infected individuals. These 20 CpG sites that were significantly associated with HIV infection were found within genes involved in immune activation (30). The most significant was 2 CpG sites located in the promoter region of NOD-like receptor family CARD domain containing 5 (*NLRC5*), an important transcriptional regulator of the Human Leucocyte Antigen (HLA) class-I genes and genes related to HLA class I antigen presentation and processing, such as *TAP1 β2M, and LMP2* (86). Hypomethylation of the 2 CpG sites (cg16411857 and cg07839457) within the promoter region of the *NLRC5* inversely correlated with viral load implying that DNA methylation of *NLRC5* is associated with HIV disease outcome (30). In a recent study, similar results were observed in HIV infected and uninfected individuals who are injectable drug users during 6-month abstinence from drug injections. HIV infection was associated with 49 differentially methylated (DM) CpG sites. The top CpG sites identified were associated with immune and viral response pathways that are associated with HIV pathogenesis, with *NRLC5* being the top-ranked gene associated with HIV status (87). Strong evidence of differential methylation within the MHC region *(HLA-F, PSORS1C2, PSORS1C3* and *Notch4*) and *NLRC5* region was also observed in children with perinatally acquired HIV. HIV was also shown to stunt B cell development and maturation *via* hypermethylation of *EBF4, FOXP1* and *DLL1* in perinatally infected children (29).

While studies on adult populations found that most DM CpG sites were hypomethylated in HIV infected individuals (30, 87), 97% of DM CpG sites tend to be hypermethylated in perinatally infected children. These differences suggest childhood acquisition of HIV alters the epigenome differently than acquisition as an adult (29). Differential methylation also occurs between perinatally infected and uninfected children (44, 88). Seeing as genetic and environmental factors influence the methylome, studies comparing the epigenetic profile of the general population is less than ideal. The use of discordant monozygotic twins with perfectly matched genetic profiles and similar lifestyles eliminates potential genetic confounders when unrelated individuals are used. Thus, variations in the methylome could be accurately attributed to exogenous factors such as viral infection (89). In a study conducted on a pair of 15-year-old monozygotic twins with discordant HIV statuses, significantly higher levels of methylated differentially methylated regions (DMRs) were observed in the infected twin compared to the uninfected sibling, further suggesting that HIV infection would cause the increase of global methylation level in perinatally infected children (44, 88). DMRs were located in chromosomes 17, 19 and 22, which are known HIV integration sites as they contain actively transcribing genes (44, 90, 91). It is possible that hypermethylation of regions in these chromosomes may be a mechanism employed by the host to suppress viral propagation. Twenty-five hyper-methylated genes in the HIV infected twin were validated at the transcriptional level. The expression of 72% of genes were downregulated by more than 50% in the HIV infected twin with *IGFBP6* and *SATB2* being the most significantly reduced genes. However, information on the role of IGFBP6 and SATB2 in HIV pathogenesis is limited (44). The use of HIV discordant monozygotic twins by Zhang et al. (44, 88) was an admirable attempt to account for the influence of genetic factors; however, it failed to account for environmental effects (44, 88). Further, only a single pair of twins were used in the study and the twins were recruited seven years after the acquisition of HIV infection. Thus, methylation changes cannot be used to distinguish between cause and consequence (44, 88).

While most studies have focused on variations in global DNA methylation among uninfected and infected individuals, the disparity has also been established in individuals with variable levels of HIV-1 viral load. Oriol-Tordera et al. (92) evaluated host genome methylation patterns of chronically HIV-1 infected individuals with high (>50,000 HIV-1-RNA copies/ml) and low (<10,000 HIV-1-RNA copies/ml) viral loads. Fifty-five DMRs were found to differentiate individuals with high viral load from those with low viral loads (92). Functional analysis showed genes involved in anti-viral activity and type I interferon γ (IFNγ) signalling to be hypermethylated in HIV infected individuals with low viral loads. Of particular interest, DMRs associated with IFNγ signalling included: *PARP9*/*DTX3L*, *MX1*, *USP18*, *IFI44L* and *PLSCR1*. In contrast, genes involved in general immune activation, such as T cell activation and differentiation, were found to be hypomethylated compared to individuals with a high HIV viral load (92). Thus, the epigenetic repression of IFNγ stimulating genes may assist in achieving control of HIV.

The studies described thus far provide valuable information on the association of aberrant methylation patterns and HIV infection at an epigenome-wide level; however, the use of whole blood, which consists of various cell types, has been used in these studies tend to be problematic. DNA methylation profiles differ strongly by cell type; therefore, variations in cell-type composition and proportions between samples can confound analysis (93). Furthermore, HIV mainly affects CD4 T cells which represents a small proportion of the tissue sampled; thus, the variation may not be detected. HIV further destroys CD4+ T cells levels; hence, measured epigenetic differences between cases and controls may only reflect differences in cell type composition and not true epigenetic differences (94). The use of homogeneous cell populations may provide a more accurate estimation of epigenome-wide methylation patterns and associated differential gene expression profiles between HIV infected and uninfected cells. CD4^+^ T lymphocytes are significant targets of HIV, with their progressive death culminating in acquired immune deficiency syndrome (AIDS). The use of the DNMT inhibitor, 5-azacytidine (5-azaC), can reverse T cell depletion, suggesting that DNA methylation may impact T cell apoptosis during HIV infection (95). Zeng et al. (96) transfected two T-cell lines (MT-2 and Jurkat cells lines) with the T-cell-tropic HIV strain, HIV-1 pNL4-3. Whole-genome methylation analysis found 1,428 hypermethylated and 1,227 hypomethylated DMRs in HIV infected MT-2 cell line compared with the uninfected controls as well as 1,231 hypermethylated and 1,833 hypomethylated DMRs in HIV infected Jurkat cells compared to uninfected control cells (96). Hypermethylated DMRs were significantly enriched in promoter and enhancer regions, suggesting that methylation changes are prone to occur in coding and transcriptional regulatory regions during HIV-1 infection (96). Hypomethylation of DMRs in 147 transcription factor binding motifs occurred in HIV infected Jurkat cells, 94 of which overlapped with the hypomethylated DMRs in the MT-2 cell line (96). HIV infected MT-2 cell lines, and Jurkat cell lines contained 83 and 53 transcription factor binding motifs found in hypermethylated DMRs. In the MT-2 cell line, five hypermethylated transcription factor binding motifs (*WT1, HIF1A, EGR1, IRF1*, and *MEF2C*) were associated with transcription factors that have been previously associated in HIV-1 induced apoptosis (96). These results suggest that the depletion of T cells during HIV infection results from aberrant DNA methylation at the binding sites of apoptosis-related transcription factors (96). Differences in epigenome-wide methylation were observed in CD4^+^ T cells isolated from individuals with varying degrees of control, suggesting that methylation status differs according to the progression of diseases state and control of infection. Furthermore, hypermethylation of TNF was characteristic in viremic individuals while *TRIM69* and *ITTGB2* were found to be hypomethylated in elite controllers (97). While the use of a homogenous *in vitro* models may provide more accurate methylation patterns, *in vitro* studies are not accurate representation of cells systems and are unable to account for ethnic differences.

Epigenome-wide characterisation reveals that global hypomethylation is prominent in HIV infected adults (30, 87), whereas global hypermethylation is prominent in HIV infected children compared to uninfected children (29, 88). Top hits include genes associated with anti-viral responses, immune defence, immune cell development and apoptosis (29, 30, 87, 96). However, the use of PBMCs and the comparison between unrelated, unmatched infected and uninfected individuals confounds results and thus, it is imperative to account for these factors. More studies should evaluate epigenetic events in monozygotic twins with discordant statuses, or a more desirable approach would be the longitudinal analysis of individuals pre- and post-HIV infection.

### Candidate Host Gene Methylation

While EWAS characterisation provides a holistic view of methylation patterns during HIV infection, it is not feasible. Thus, many researchers opt for a targeted approach by analysing the epigenetic regulation of specific genes. The four most common techniques used to determine the methylation status of specific CpG sites includes: (i) methylation-specific restriction endonucleases (MSRE) followed by qPCR using primers surrounding the sequence of interest, (ii) pyrosequencing, (iii) methylation-specific high-resolution DNA melting analysis and (iv) quantitative methylation-specific polymerase chain reaction (98). Several studies have investigated the effect of HIV infection on specific HIV associated genes.

The surface expression of C-C chemokine receptor type 5 (CCR5) influences HIV-1 acquisition and disease progression by facilitating HIV-1 viral entry into T cells (99, 100). A common determinant of *CCR5* expression is specific polymorphisms in open reading frames and cis-regulatory regions of *CCR5* (101). One such polymorphism is a 32 base pair deletion in the open reading frame of *CCR5* (CCR5-Δ32). Individuals homozygous for the CCR5-Δ32 mutation cannot produce complete CCR5 proteins; thus, their T cells surface is devoid of the receptor, providing them with protection against HIV (102, 103). However, polymorphisms do not account for the variation in CCR5 expression between subsets of T cells and altered expression upon T cell activation (104–106). *In vivo* and *ex vivo* analysis by Gornalusse et al. (107) showed that methylation levels within the *CCR5* gene might account for these variations (107). Sorted T cells with higher methylation content within the *cis*-region of *CCR-5* correlated with low CCR5 surface levels. CpG sites in the regulatory region of *CCR5* were mostly methylated in naïve T cells, whereas hypomethylation was prevalent in memory T cells (107). *In vitro* activation of naïve T cells was associated with demethylation of *CCR5* and concomitant increase in *CCR5* expression. These results were confirmed in a cohort of individuals with primary HIV infection and two cohorts of individuals with untreated chronic infection. However, viral load suppression during ART was associated with increased methylation in *CCR5*-*cis* regions and low CCR5 levels during primary infection (107). Furthermore, the authors demonstrated that specific CCR5 haplotypes contain polymorphism, which may remove CpG sites, resulting in cis-regions resistant to undergoing activation-induced demethylation and are thus constitutively expressed. Therefore, CCR5 surface levels and HIV susceptibility depend on both genetic and epigenetic mechanisms (107).

Genetic variations in the HLA region are known to influence host control of HIV infection (108, 109). HLA molecules present intracellularly derived peptides to immune cells, which elicits immune response upon recognising pathogenic peptides (110). Several previously discussed EWAS have identified differential methylation within the HLA loci in HIV positive individuals (29, 86). The elevated levels of the class I HLA-A molecules are associated with higher HIV viral load and poor HIV control. In contrast, low expression of HLA-A is associated with improved control of viremia and slower progression to AIDS (111). Methylation of the HLA-A promoter results in the reduced expression of HLA-A (112). Moreover, allelic lineage-specific methylation patterns within the HLA-A promoter region are inversely related to HLA expression. Increased DNA methylation levels correlated significantly with reduced HLA-A expression levels (112). Gross et al. (26) found that an entire HLA locus had notably reduced methylation levels in HIV infected individuals compared to uninfected individuals (26). Furthermore, several differentially methylated markers were found surrounding a single nucleotide polymorphism (SNP), rs2395029, within the HLA region (26). This variant is predictive for the presence of HLA-B*5701 and is common in HIV positive non-progressors. Further examination of this locus in neutrophils and CD4^+^ T cells found that the gene body of *HLA Complex P5 (HCP5)* was differentially methylated in neutrophils, and the methylation level of *HCP5* correlated with CD4^+^:CD8^+^ T cell ratio (26). Thus, methylation dynamics plays a critical role in HIV control through its regulation of the HLA system (26, 111, 112).

A specialised subset of CD4 T lymphocytes known as regulatory T cells or T_regs_ plays an essential role in suppressing hyperactive immune responses that may occur during the course of HIV infection (113). However, T_regs_ are also susceptible to HIV infection as they contain receptors that participate in viral entry (114, 115). The maintenance of T_reg_ functioning is heavily dependent on the surface expression of Forkhead Box Protein 3 (FOXP3) (116). *In vitro* transfection of T_regs_ with HIV-1 was shown to impair Treg functioning through the methylation of CpG sites found in *FOX3P* regulatory regions (41). However, *in vivo* analysis of *FOXP3* promoters from T*regs* isolated from PBMCs and colon mucosa of chronic HIV infected patients was demethylated, resulting in the increased expression of *FOX3P* (117). In both studies, *FOX3P* promoter methylation was associated with altered levels of DNA methylation regulating enzymes (41). High levels of DNMT3B were associated with the elevated methylation in the *in vitro* study while a significant reduction in DNMT1, DMAP1, METTL7B, and METTL1 was responsible for the reduced methylation in the *in vivo* study (117).

DNMTs were also shown to influence interferon-gamma (IFNγ) levels (38). INFγ, a cytokine produced by type 1 T helper cells, CD8+ cytotoxic T cells and natural killer cells, facilitates inflammation and regulates antigen presentation and macrophage differentiation upon viral infections (80). High levels of DNMTs in HIV infected T helper cells were shown to induce methylation at the SnaBI site in *INFγ* promoters resulting in low levels on *IFNγ* (38) The aberrant expression is due to methylation silencing and may play a role in the gradual loss of type 1 helper cell response seen in AIDS patients.

HIV positive women have an increased risk of developing cervical cancer and precursor lesions [cervical intraepithelial neoplasia (CIN)] (118–120). Hypermethylation and subsequent silencing of tumour suppressor genes result in gene silencing and represents an essential step for cervical cancer development (121, 122). Methylation levels of the tumour suppressor *EPB41L3* were significantly higher in HIV seropositive women with moderate grade neoplasia compared to HIV seronegative women (123). Methylation levels of microRNA-124–2 (miR-124–2), was significantly associated with HIV positive women with low, moderate and severe grade neoplasia compared to HIV negative women (124); however, no association was found between the methylation content of the tumour suppressor genes *CADM1, MAL RARB, DAPK1* and *PAX1* in HIV (124, 125). The methylation of *ASCL1, LHX8* and *ST6GALNAC5* was significantly higher in HIV seropositive women with low to moderate grade neoplasia than HIV seronegative women. However, methylation levels were comparable between HIV seropositive and HIV seronegative women with high-grade neoplasia (126).

Most recently, Singh et al. (127) found that methylation levels within the gene promoter of the host anti-viral restriction factor, *bone marrow stromal cell antigen 2* (*BST2* or *tetherin*) was associated with BST2 expression and HIV disease state. Methylation levels were significantly elevated in all nine CpG sites within HIV infected individuals compared to the uninfected group. Within the HIV positive group, CpG promoter methylation of *BST2* was further evaluated across four different time points (pre-infection, 3-months. 12-months and 36-months post-infection). An inverse correlation between *BST2* methylation and expression was observed at all time points. Furthermore, in an *in vitro* HIV replication assay, treatment with the DNA hypomethylation drug, 5’-Aza-CdR corresponded with an increased expression of *BST2* and lower viral load, suggesting that controlling regulation may be an important strategy in controlling HIV infection (127).

**Figure 2:**
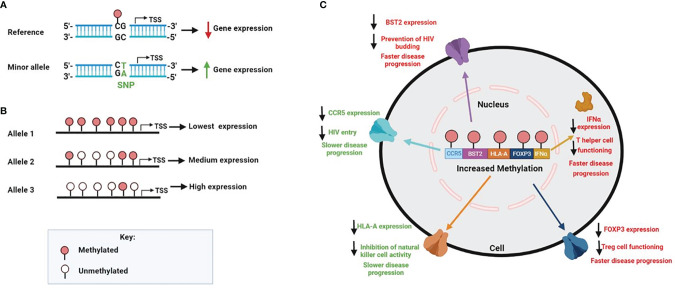
Factors that contribute to human genomic methylation on HIV disease. **(A)** SNPs found in regulatory gene regions can create or abolish CPG sites, which in turn may affect methylation and gene expression. In the genomic sequence, if C is followed by a G, the C can be methylated; however, when the SNP is mutated from a G to a T, it removes the CpG site and methylation cannot occur **(B)** DNA methylation can also differ among alleles of a given gene. The number of methylated CpG sites on each allele can affect expression accordingly **(C)** Increased promoter methylation generally results in decreased mRNA gene expression, which is specifically observed within the HIV setting for the following genes CCR5, BST2, HLA-A, FOX3P and IFNα. Since expression variability of these genes are directly linked to HIV pathogenesis, a change in methylation levels have shown to alter HIV disease progression. Higher levels of methylation for *CCR5* and *HLA*-A results in slower HIV disease progression, however, higher levels of methylation for *BST2, FOX3P* and *IFNα* results in faster disease progression.

While DNA methylation is an epigenetic modification, candidate gene methylation may be influenced by variations in the DNA sequence. Several studies have mapped the interactions between genetic differences and variations in DNA methylation across numerous tissue and cell types (128–131). The methylation quantitative trait loci showed that up to 48% of inter-individual variation in DNA methylation was related to CpG sites that were associated with nearby single nucleotide polymorphisms (SNPs) found in *cis* regulatory regions (132, 133). SNPs located near or in CpG sites found in the promoter region of genes can either produce or remove CpG site methylation, leading to an alteration in the expression of the genes (**Figure 2A**). DNA methylation can also differ among alleles of a given gene. This is referred to allele-specific methylation (**Figure 2B**). For example, the promoter region of HLA-A*24 (highest HLA-A expressing lineage) and -A*03 (lowest HLA-A expressing linage*)* contain a similar number of CpG sites; however, only one CpG site was found methylated in the promoter of the HLA-A*24 lineage, while most CpG sites were found to be methylated in the HLA-A*03 linage (112). The influence of genetic variation on promoter methylation of specific host genes in relation to HIV pathogenesis has yet to be investigated. As discussed in this section, we, however, do know that increased promoter methylation generally lowers mRNA expression of specific genes affecting HIV disease progression (**Figure 2C**).

## Methylation Controlled Host Genes Observed in Other Diseases and Models

With only a few studies evaluating the influence of HIV on the methylation of specific host genes, further examination is essential (26, 38, 41, 107, 117, 123, 125, 126). Henceforth, we discuss potential host genes whose methylation status should be investigated with regard to HIV. These genes have been previously shown to associate with HIV disease and were shown to be controlled by DNA methylation in conditions other than HIV. Based on the principle that these genes have been regulated by DNA methylation for a particular disease association, we assume that they may also be regulated similarly in an HIV setting.

For instance, the co-receptor C-X-C chemokine receptor type 4 (CXCR4), like CCR5, mediates the entry of HIV into host cells. Low surface expression of CXCR4 confers with reduced viral entry, while increased expression is associated with the elevated viral entry. Therefore alternations of CXCR4 expression has a significant influence on HIV progression (134, 135). DNA methylation has been shown to regulate CXCR4 expression in pancreatic cancer (136), sporadic breast cancer (137), and primary myelofibrosis (138).

Another example is the host restriction factor, sterile alpha motif and histidine/aspartic acid domain-containing protein 1 (SAMHD1) which limits HIV reverse transcription by depleting the intracellular pool of deoxynucleotide triphosphates (139, 140). De Silva et al. (141) used CD4^+^ T cell lines as a model to identify mechanisms that regulate *SAMHD1* gene expression. The results indicated that the *SAMHD1* promoter contains a CpG island proximal to the initiation codon of the *SAMHD1* gene, which, upon DNA methylation, leads to transcriptional repression in certain CD4^+^ T cell lines (142). Regarding disease association, reduced levels of *SAMHD1* expression corresponded with *SAMHD1* promoter methylation in lung cancer (143) and patients with Sezary syndrome (141).

The tumour suppressor, p53 and its downstream gene, *p21*, were shown to hinder early-stage replication of HIV-1 (144). p21, a cyclin dependant kinase, promotes cell cycle arrest by downregulating G1/S transition (144, 145). p21 is also shown to regulate SAMHD1 in HIV-1 infection (145). Epigenetic alterations, including promoter DNA methylation and histone deacetylation, have long been established as crucial mechanisms of carcinogenesis (146–148). p53 promoter methylation leads to downregulation of p53 in several cancers (149–151). Loss of p21 has been shown to occur in colorectal cancer (152). Additionally, the p21 gene is frequently methylated and is an essential factor in predicting the clinical outcome of acute lymphoblastic leukaemia patients (153). The loss of p21 expression was commonly observed in lung cancer and malignant pleural mesothelioma, and aberrant methylation was one of the mechanisms of suppression of p21 (154).

Methylation of several other host factors such as CCR2, CCL2, CXCR6, CCL5, TSG101, PD-1, PD-L1, TIM3, LAG-3, CTLA-4, TRIM22, DC-SIGN (CD209), IL-10, IL-32, IRF1, Perforin, ICAM-1, and PCSK9 could potentially play a role in HIV disease. **Table 1** provides a list of host factors associated with HIV pathogenesis which should be examined in future methylation studies. Although these disease-methylation associations have been shown in other diseases, it is yet to be proven in HIV disease. Based on the principle that these genes have been regulated by DNA methylation for a particular disease association, we assume that they may also be regulated similarly in an HIV setting. These listed genes may be potential host gene targets that may provide an alternative approach towards precision medicine or personalised therapeutic interventions against HIV and other diseases.

**Table 1:** HIV-associated host genes that are regulated by methylation in other diseases or *in vitro* models.

Gene	Role in HIV-1 pathogenesis	Citation	Disease or *in vitro* models in which DNA methylation is established	Citation
***Viral entry***				
***CXCR4***	Facilitates viral entry	(134, 135)	Pancreatic cancer, Sporadic breast cancer, and primary myelofibrosis	(136–138)
***CCR2***	Minor HIV co-receptor which mediates viral entry	(155, 156)	Human monocytic cells	(157)
***CCL2***	A ligand of CCR2 which upregulates CXCR4 expression on CD4+ T cells, thus facilitating viral entry. Facilitates transmigration of HIV infected leukocytes across the blood-brain barrier	(158, 159)	Gout, Small cell lung cancer, Raw264.7 macrophages	(160–162)
***CXCR6***	HIV co-receptor which mediates viral entry	(163, 164)	Hepatosplenic T–cell lymphoma, and Systemic Sclerosis	(165, 166)
***CCL5 (RANTES)***	Ligand for CCR5. It suppresses infection of R5 strains of HIV-1 by blocking CCR5	(167, 168)	Ageing and childhood obesity-associated asthma	(169, 170)
***HIV restriction factor***				
***SAMHD1***	Restricts HIV replication	(139, 140)	Lung cancer and Sezary syndrome	(142, 143)
***P53***	Restricts HIV replication	(144)	Ovarian cancer, breast cancer, hepatocellular carcinoma and colon cancer	(149–151)
***p21***	Restricts HIV replication	(144, 145)	Colorectal cancer, lung cancer and malignant pleural mesothelioma and acute lymphoblastic leukemia	(152–154)
***TSG101***	Inhibits HIV budding	(171)	Cervical cancer	(172)
***Immune checkpoint molecules***				
***PD-1***	Immune checkpoint molecule expressed on exhausted T cells, inhibit productive HIV infection, thereby facilitating the establishment of latent HIV infection.	(173, 174)	Colorectal cancer, breast cancer, head and neck squamous cell carcinoma, myelodysplastic syndrome and prostate cancer	(175–179)
***PD-L1***	Ligand for PD-1. Immune checkpoint molecule expressed on exhausted T cells, inhibit productive HIV infection, thereby facilitating the establishment of latent HIV infection.	(180)	Colorectal cancer, Non-small-cell lung carcinoma, and acute myeloid leukaemia	(175, 181, 182)
***TIM3***	Suppress effector functions of activated T cells in chronic uncontrolled viral infection with HIV-1.	(183)	Colorectal cancer, breast cancer and gastric cancer	(175, 179, 184)
***LAG-3***	Immune checkpoint molecule, induces immune exhaustion and facilitates HIV latency	(185, 186)	Colorectal cancer, breast cancer, clear cell renal cell carcinoma, melanoma	(175, 179, 187, 188)
***CTLA-4***	Downregulates T cell functioning and associated with HIV disease progression	(189)	Colorectal cancer, breast cancer, rheumatoid arthritis, myasthenia gravis, head and neck squamous cell carcinomas	(175, 179, 190–192)
**Other**				
**TRIM22**	Inhibits HIV transcription and promotes HIV latency	(193)	Hepatitis B virus, Systemic lupus erythematosus	(194, 195)
**DC-SIGN (CD209)**	Recpetor found on dendritic cells which binds to gp120 of HIV and facilitate the dissemination of HIV	(196, 197)	Dendritic cells	(198)
**IL-10**	Increases post-HIV infection by inhibiting HIV-1 specific T-cell responses	(199)	Rheumathoid arthritis, Behçet’s disease	(200, 201)
**IL-32**	Induces hostile cytokine environment that hinders HIV fusion and replication	(202, 203)	Hek293 (*in vitro*), Juvenile idiopathic arthritis, Influenza A	(204–206)
**IRF1**	activating the transcription of HIV genome during the early stage of HIV replication	(207, 208)	Paediatric obstructive sleep apnea	(209)
**Perforin**	Associated with slow HIV progression. Mediates the killing of HIV-infected cells by CD8+ T-cells	(210, 211)	CD4 and CD8 T cells, systemic lupus erythematosus, chronic fatigue syndrome, multiple sclerosis	(18, 212–214)
**ICAM-1**	promotes HIV-mediated syncytia formation and viral spread.	(215)	Autoimmune thyroid diseases, and primary bladder carcinoma.	(216, 217)
**PCSK9**	Mediates HIV-Associated Dyslipidemia		Coronary artery disease, Congenital Aortic Valve Stenosis Type 2 Diabetes and Metabolic Syndrome	(218–220)

## DNA Methylation: A Valuable Tool for Epi-Therapeutics and Precision Medicine

‘The Berlin patient’ and ‘the London patient’ were the first two individuals reportedly “cured” of HIV. They both received a stem cell transplant containing the CCR5 Δ-32 mutation to treat their leukaemia which consequentially eliminated the virus from their bodies (221, 222). Such cases provided proof that HIV-1 can be eradicated in those already living with the virus. Given that this approach is not feasible for most people living with HIV, other therapeutic strategies are essential. Furthermore, recent studies have shown that early treatment with ART, is ineffective against returning the altered DNA methylation profile of HIV positive individuals during acute infection (223). Therefore, there is a need for epigenetic strategies for the treatment of HIV.

Recently, Shrivastava et al. (224) developed a zinc finger protein (ZFP-362) that specifically targeted the HIV-1 promoter region. The ZFP-362 fuses to active domains of DNMT3A and induces a long-term stable epigenetic repression of HIV-1. This suppression was found to be driven by DNA methylation (224). Like ART, this intervention may repress viral transcription and control viral replication in HIV positive individuals; however, it is ineffective against latent HIV reservoirs. Thus, efforts have mainly been focused on targeting the latent HIV-1 reservoir responsible for viral persistence and strengthening immunological defences against HIV. Many researchers are adopting the “shock and kill” approach to targeting HIV. This strategy involves the forced reversal of HIV latency (shock) followed by the robust elimination of infected cells by viral or host immune-mediated cytolysis (kill). Therefore novel approaches for the development of latency-reversing agents (LRA) are needed (225). Much interest has been given to the development of epi-LRA – agents that disrupt latency by interfering with the epigenetic silencing mechanism of the 5’LTR (226). In the instance of methylation of 5’LTR, the use of DNMT inhibitors have been considered (31).

Bouchat et al. (227) found that the DNMT inhibitor, 5‐AzaC, combined with histone deacetylase inhibitors panobinostat or romidepsin, was potent in reducing HIV-1 latent reservoirs in ART-treated patients (227). The 5-AzaC analogue, 5-aza-2′ deoxycytidine (5‐AzadC), alone and in combination with TNFα and prostratin, significantly increased HIV gene expression through altered methylation levels (31, 227). Both 5-AzaC and 5-AzadC, commercially known as Vidaza^®^ and Dacogen^®^, respectively, have been approved by the FDA to treat myelodysplastic syndrome and in phase II clinical trials for chronic myelomonocytic leukaemia (227–229). Treatment with either 5-AzaC or 5-AzadC was shown to increase the overall survival of patients with higher-risk myelodysplastic syndromes and prolong time to leukaemia transformation and death compared to conventional care regimens (230–232). According to clinicaltrials.gov, 389 clinical trials are actively investigating 5-AzaC and 5-AzadC as interventions for various cancers and conditions. These include: ependymoma, breast cancers, lymphomas, osteosarcoma, and pancreatic cancer, as well as other conditions such as immune thrombocytopenia, sickle cell disease, myelofibrosis, and COVID-19. Therefore, the inclusion of DNMT inhibitors with ART could represent a significant step towards the elimination of the latent HIV-1 reservoir and clearance of virus from infected patients.

Other novel technologies, such as Clustered Regularly Interspaced Short Palindromic Repeats (CRISPR), have great potential in eradicating viral genomes from infected individuals by editing genes as well as the methylation levels associated with HIV. Ebina et al. (233) successfully excised the latently integrated provirus from the host genome and restricted transcriptionally active provirus using the CRISPR/Cas9 approach (233). CRISPR-Cas9 editing of the host genome has also been investigated as an intervention against HIV. Silencing of *CCR5* and *CXCR4* genes by CRISPR have already been shown as effective towards a functional cure for HIV-1 infection (234–236). While the conventional CRISPR approach may have revolutionised genetic therapies, it permanently switches off host genes and may have unwanted consequences such as off-target gene mutations (237, 238). Therefore, approaches that edit the epigenome rather than the genome may be a more suitable and safer strategy. CRISPR-based epigenome technologies involve the fusion of inactivated Cas9 (dCas9) with DNA methyltransferase or demethylase enzymes, allowing for manipulating methylation levels at specific CpG sites. Because this approach targets the epigenome and uses inactivated Cas9, it will enable reversible editing and prevents the formation of double-strand breaks (239–241). Therefore, this approach may be ideal in prospective studies that evaluate host gene regulation as a treatment strategy against HIV (240).

As the medical field rapidly moves towards precision medicine and theragnostic approaches, DNA methylation profiling can play a tremendous role in these strategies. DNA methylations can serve as biomarkers for diagnosis, prognosis, monitoring and predicting treatment response and disease outcome (242). Due to its dynamic and stable nature, it is more reliable and suitable than genetic and protein-based biomarkers. Methylation levels can be easily measured in circulating cell-free DNA, which is the preferable method in clinical settings as it is minimally invasive (243). Several DNA methylation-based *in vitro* diagnostic tests have been developed and commercialised for profiling DNA methylation (241). Tests may be specific for a disease such as Epi proColon^®^ 2.0 CE, which detects methylated *Septin9* to diagnose colon cancer and Bladder EpiCheck^®^, which measures changes in methylation of 15 genes associated with bladder cancer (244, 245). The utilisation of the EpiSign assay has been well established in clinical diagnostic laboratories and uses genome-wide methylation patterns to diagnose up to 42 rare neurodevelopmental Mendelian syndromes (246, 247). Many of the commercialised clinical DNA methylation assays implement practical and cost-effective assays such as qPCR and microarrays. The use of DNA methylation-based biomarkers for precision medicine has been extensively studied with regards to cancer; however, its application has great potential in other diseases, including HIV. For instance, DNA methylation has been shown to be a potentially effective prognostic biomarker for predicting risk and type of HIV-associated lymphomas and HIV associated cognitive impairment; however, these results are yet to be translated to a clinical setting (94, 248). There is still a lot to be investigated regarding the epigenetic signature of HIV for precision medicine. Future studies should focus on using well-characterised clinical cohorts to evaluate methylation profiling as a biomarker for predicting HIV disease course, development of HIV associated comorbidities, monitoring patient response to ARVs and personalised therapy.

The Epi-therapeutic interventions, either through LRA or CRISPR technologies and DNA methylation in precision medicine and theragnostics, provides a novel and powerful approach against HIV. However, there is much-needed research to be done to translate these approaches into a clinical setting.

## Conclusion and Future Perspectives

Since the beginning of the HIV epidemic, the impact of host genetic variations on HIV susceptibility and disease outcomes has attracted a vast amount of attention, while epigenetic changes have long been neglected. This review provided a comprehensive overview of the intricate interplay between DNA methylation and viral and host genome. Once integrated, the HIV viral genome is subject to the intense epigenetic environment of the host genome. This includes silencing of HIV transcription *via* DNA methylation. Integration of the proviral genome also induces aberrant methylation of the host genome, influencing HIV disease progression. Several host genes involved in viral entry, anti-viral responses and immune defences are altered by DNA methylation in HIV infected individuals.

However, many of the studies discussed are limited by the study designs used. Many of the studies discussed failed to account for the influence of genetic and/or environmental factors on promoter methylation. Another drawback of most studies reviewed is the type of sample that was used. The type of sample selected for a study involving DNA methylation is crucial as methylation patterns differ substantially according to cell type (93). Studies using mixed cell samples such as whole blood or PBMCs need to account for cell type composition and variation in the methylation patterns of different cells. Some studies have tried to account for account for cell type heterogeneity by transfecting homogenous T cell lines (95–97). However *in vitro* studies are not accurate representation of cells systems and are unable to account for ethnic differences. Increased susceptibility to HIV and varying responses to ARVs have been noted amongst different ethnic groups [extensively reviewed in (249)]. Disparities regarding DNA methylation have also been observed between diverse ethnic populations, including Caucasians, Hispanics, Middle Eastern, and African populations and may serve as a biomarker for underlying ethnic health disparities between human populations (250). Thus far, very little is known about the contribution of DNA methylation on ethnic differences to HIV acquisition, disease and treatment outcomes. Seeing that aberrant methylation patterns have been associated with HIV and that the rate of incidence differs amongst different ethnic groups, it is vital ethnic differences are taken into consideration when conducting studies and clinical trials therefore researchers should also take ethnicity into consideration (249, 250). The results of trials on one ethnic group may not necessarily be applicable to another ethnic, therefore researchers should also take ethnicity into consideration. We believe that the ideal model for epigenetic studies related to HIV disease are sorted PBMCs or CD4+ T cells that are isolated form a prospectively obtained longitudinal cohort consisting of different ethnic groups. Admittedly, it will be challenging to recruit and maintain such a cohort, nonetheless, more accurate and useful information can be gained from such a study design.

There is still a lot of gaps in knowledge regarding the relationship between methylation and HIV. But once we have a complete picture, the knowledge gained will contribute substantially to understanding HIV disease. Moreover, the use of epigenetic interventions such as DNMTs inhibitors as LRA, CRISPR editing, and methylation biomarkers may revolutionise our fight against HIV and the AIDS pandemic.

## Author Contributions

Conceptualization and conceiving of idea, VR. Additional input with regards to conceptualization, ThiA. Writing, ThiA, TheA, and UR. Research, ThiA, TheA, UR, and RC. Figure design, ThiA. Editing of manuscript, VR. All authors contributed to the article and approved the submitted version.

## Funding

This publication was supported by the South African Medical Research Council with funds received from the South African Department of Science and Technology. VR was funded as a FLAIR Research Fellow [the Future Leader in African Independent Research (FLAIR) Fellowship Programme was a partnership between the African Academy of Sciences (AAS) and the Royal Society that was funded by the UK Government as part of the Global Challenge Research Fund (GCRF) Grant # FLAIR-FLR\R1\190204]; supported by the South African Medical Research Council (SAMRC) with funds from the Department of Science and Technology (DST); and VR was also supported in part through the Sub-Saharan African Network for TB/HIV Research Excellence (SANTHE), a DELTAS Africa Initiative (Grant # DEL-15-006) by the AAS.

## Conflict of Interest

The authors declare that the research was conducted in the absence of any commercial or financial relationships that could be construed as a potential conflict of interest.

## Publisher’s Note

All claims expressed in this article are solely those of the authors and do not necessarily represent those of their affiliated organizations, or those of the publisher, the editors and the reviewers. Any product that may be evaluated in this article, or claim that may be made by its manufacturer, is not guaranteed or endorsed by the publisher.
